# Generation of convergent light beams by using surface plasmon locked Smith-Purcell radiation

**DOI:** 10.1038/s41598-017-11622-1

**Published:** 2017-09-11

**Authors:** Yi-Chieh Lai, Tzu Cheng Kuang, Bo Han Cheng, Yung-Chiang Lan, Din Ping Tsai

**Affiliations:** 1 Department of Photonics and Advanced Optoelectronic Technology Center, National Cheng Kung University, Tainan, 701 Taiwan; 2 Research Center for Applied Sciences, Academia Sinica, Taipei, 115 Taiwan; 3 Department of Physics, National Taiwan University, Taipei, 10617 Taiwan

## Abstract

An electron bunch passing through a periodic metal grating can emit Smith-Purcell radiation (SPR). Recently, it has been found that SPR can be locked and enhanced at some emission wavelength and angle by excitation of surface plasmon (SP) on the metal substrate. In this work, the generation of a convergent light beam via using the SP-locked SPR is proposed and investigated by computer simulations. The proposed structure is composed of an insulator-metal-insulator (IMI) substrate with chirped gratings on the substrate. The chirped gratings are designed such that a convergent beam containing a single wavelength is formed directly above the gratings when an electron bunch passes beneath the substrate. The wavelength of the convergent beam changes with the refractive index of dielectric layer of the IMI structure, which is determined by the frequency of SP on the IMI substrate excited by the electron bunch. Moreover, reversing the direction of electron bunch will make the emitted light from the proposed structure to switch from a convergent beam to a divergent beam. Finally, the formation of a convergent beam containing red, green and blue lights just above the chirped gratings is also demonstrated. This work offers potential applications in the fields of optical imaging, optical beam steering, holography, microdisplay, cryptography and light source.

## Introduction

Optical beam steering is an important issue in nanoscale imaging^1^. Recently, plasmonic lens that uses a metal film with a nanoslit surrounding by a few pairs of chirped nanogrooves at the output surface to achieve nanoscale beam focusing has been proposed and demonstrated^2–4^. In this device, the incident beam will couple into surface plasmons (SPs) in the nanoslit (i.e. a metal-insulator-metal (MIM) structure)^5^. At the output surface, SPs will be transformed into radiation and scattered into the nanogrooves which will also be transformed into radiation with different phases and amplitudes. Both the radiations from the nanoslit and nanogrooves shape the output beam. Very recently, the side-illuminated plasmonic lens that strongly depends on the transverse propagation of SPs in the MIM waveguide is also considered^6^. However, all of the plasmonic lenses suffer from some drawbacks such as lacking of frequency tunability and incapability to generate a focusing beam with multiple frequencies.

As an electron bunch pass above a periodic metal grating, it is capable of generating far-field radiation owing to the coherent oscillation of free and imaged charges on the grating, i.e. the so-called Smith-Purcell radiation (SPR)^7–13^. Because the electron bunch carries wide frequency information, the emission frequency of SPR can range from millimeter to visible light which is determined by the grating period. Especially, the SPR-based terahertz (THz) light source has drawn a lot of attention in recent research^14–17^. Very recently, it has been found that SPR can be locked and enhanced at some emission wavelength and angle by excitation of SPs on the substrate^18,19^. This phenomenon is attributed to that the energy from electron concentrated in the excited SPs and then transformed into radiation via SPR mechanism.

SPs on an insulator-metal-insulator (IMI) structure can also be excited by an electron bunch passing above it^5,20,21^. The frequency of SPs that is determined from the intersection points of the dispersive curves of SP and electron bunch can be altered by changing the relative permittivity of the insulator. Combining the frequency adjustable IMI structure and SP-locked SPR with a specific emission angle, the nanoscale convergent beam containing a single wavelength or multiple wavelengths can be yielded. However, this kind of device has never been explored before. In this work, the SP-locked SPR with chirped gratings on an IMI substrate for generation of a convergent beam is proposed and investigated by finite-difference time domain (FDTD) simulations. First, a single-wavelength convergent beam emitted from an electron bunch passing beneath the proposed structure is examined. Next, reversing the electron bunch moving direction to make the beam become divergent is also demonstrated. Finally, the formation of a convergent beam composed of red, green and blue lights by using the proposed structure is investigated.

## Results

**Figure 1:**
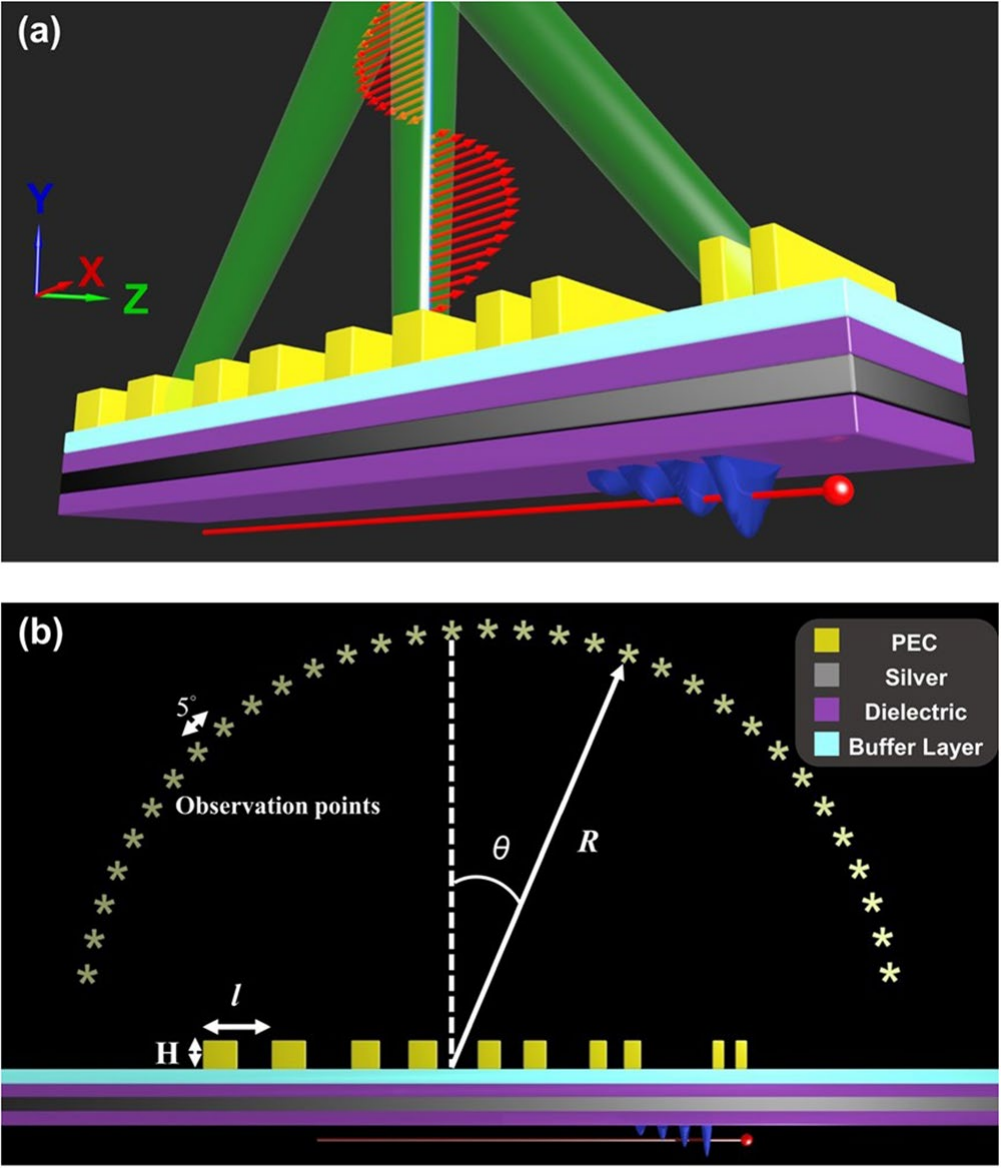
Schematic diagram and simulation model for generation of convergent beams. (**a**) Schematic diagram of a convergent beam generated by SP-locked SPR with chirped gratings on an IMI substrate. The electron bunch passes beneath the IMI substrate to excite SP. (**b**) Two-dimensional simulation model. The observation points lie on the circumference of upper semicircle of radius *R* = 4 μm and are distributed for every 5°.

Figure 1(a) plots the schematic diagram of a convergent beam containing a single wavelength generated by the SP-locked SPR with chirped gratings. In Fig. 1(a), the substrate is an IMI structure formed of a silver (Ag) film (the gray layer) sandwiched between two dielectric films with the same refractive index (the purple layers). The thicknesses of the silver film and the dielectric films are all 20 nm. A buffer layer (the light blue layer) with a refractive index closed to air (here set to 1.1) and 20 nm thick is added for SPs not to be destroyed by the gratings. The perfect electrical conductor (PEC) chirped gratings are deposited on the buffer layer with the grating period *l* changed (along the positive x-direction) from 185 to 125 nm, from 216 to 148 nm and from 256 to 174 nm for the designed radiation wavelengths of 457 (blue), 535 (green) and 633 nm (red), respectively. In our structure, every two grooves have the same period. And the depth (H) and width of the groove are equal to 60 nm and *l*/2, respectively. The detailed values of *l* for each wavelength are listed in Table 1 (see Method and Materials). (The design methodology is to adjust the period of groove according to its position and required emission angle). For SP-locked SPR with emission wavelengths of 633 nm, 535 nm and 457 nm, the refractive indices (n) of the dielectric films are set as 2.6, 2.1 and 1.7, respectively. The total lengths of the IMI structure and the buffer layer are both 10 μm. (The chirped gratings are placed at the center of the substrate).

**Table 1:** Designed periods of chirped gratings for generation of a convergent beam containing a single wavelength.

Wavelength	Designed values (*l*) of groove period (along positive x-direction) (nm)
457 nm	185, 182, 177, 173, 169, 164, 160, 156, 152, 148, 145, 142, 139, 136, 134, 132, 130, 129, 127, 126, 125 (total 21 values)
535 nm	216, 210, 204, 198, 192, 187, 181, 176, 171, 166, 163, 159, 156, 154, 151, 149, 147 (total 17 values)
633 nm	256, 248, 240, 232, 223, 215, 208, 201, 195, 190, 186, 182, 179, 176, 174 (total 15 values)

The electron bunch moves along the positive x-direction and is beneath the IMI structure with the distance between the lower dielectric layer and the electron bunch to be 10 nm. (Hence, the SPR is TM polarized). The electron energy (*E*) is 30 keV (i.e. the relativistic factor of electron β = 0.328). The observation points lie on the circumference of upper semicircle of radius *R* = 4 μm and distributed for every 5° as presented in Fig. 1(b). The convergent spot is designed directly above the gratings (i.e. *R* = 4 μm and θ = 0°, see Fig. 1(b)). And the background material is assumed to be air. The simulation method and detailed settings are given in the section of Method and Materials. The plasma and collision frequencies of Drude model of Ag are also shown in Method and Materials. Notably, the silver film can be replaced by other noble metals such as gold or aluminum which relies on the designed radiation wavelength band of the convergent beam. And the real metal gratings can be substituted for the PEC gratings. (Our simulation results indicate that using Ag gratings instead of PEC grating doesn’t affect the convergent beam formation).

**Figure 2:**
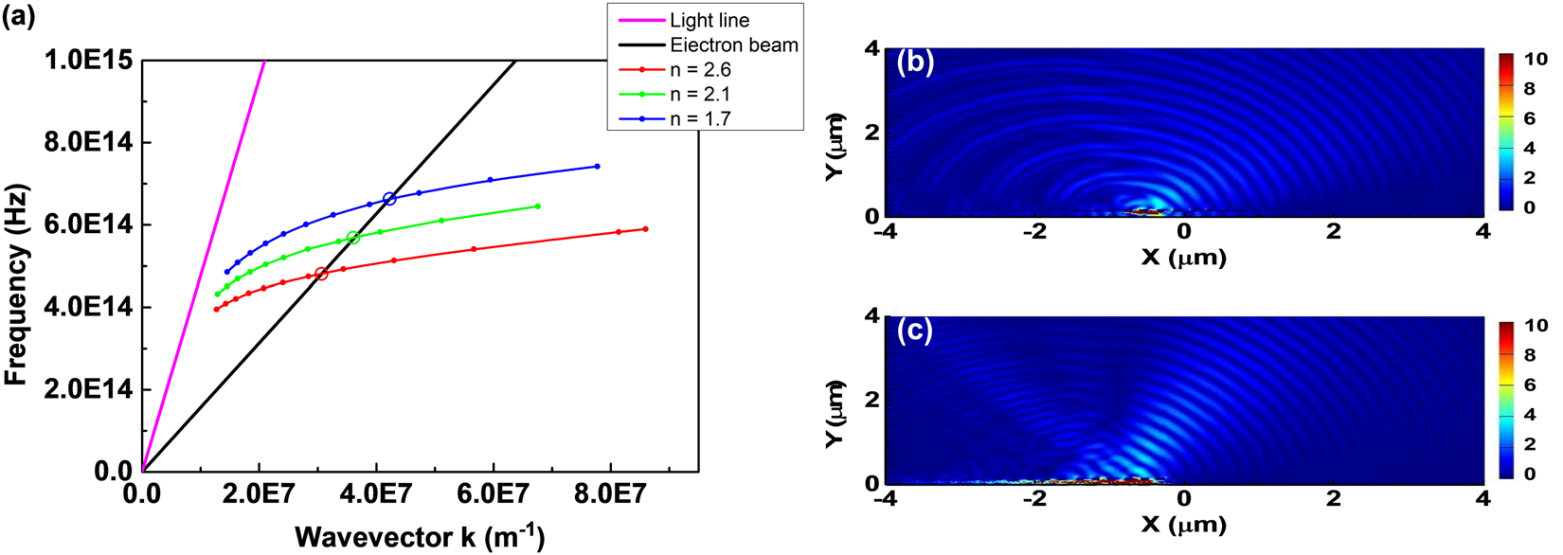
Dispersion curves of SP and traditional and SP-locked SPRs. (**a**) Dispersion curves of SP on IMI substrate for refractive indices (n) equal to 2.6 (red), 2.1 (green) and 1.7 (blue), electron bunch with *E* = 30 keV (black) and light line in vacuum (pink). (**b**) Simulated contours of |Hz| of traditional SPR for electron bunch moving under uniform PEC gratings with *l* = 228 nm. (**c**) Simulated contours of |Hz| of SP-locked SPR for electron bunch moving under proposed structure in Fig. 1 except for uniform PEC gratings with *l* = 228 nm. In (**b**) and (**c**), the distance between electron bunch and structure is 10 nm.

The property of SPs on the IMI substrate of Fig. 1(a) excited by an electron bunch is examined first. Figure 2(a) plots the dispersive curves of SPs on the IMI structure for three different refractive indices of the dielectric layers, electron bunch with *E* = 30 keV (obtained from ω = *kv* and *v* is the electron bunch velocity) and light line in vacuum. In Fig. 2(a), the cross points of the dispersive curves provide the frequencies and wavevectors of the SPs excited by the electron bunch. Figure 2(a) also shows that the frequency of the electron-excited SPs decreases with increasing the refractive index of dielectric layer. For an electron bunch passing beneath a metallic grating, the relationship between the emission wavelength and angle of SPR can be express as^10^

1 $$\lambda =\frac{{l}}{|m|}({\beta }^{-1}-\,\sin \,\theta )$$

where λ is the emission wavelength and β is the relativistic factor; θ denotes the observation angle measured from the direction normal to the grating surface (θ > 0 (θ < 0) for forward (backward) emission, see Fig. 1(b)); *l* is the period of grating and *m* denotes the order of SPR. Equation (1) reveals that SPR emits a continuous spectrum with the emission angle ranging from −90° to 90°. Conversely for SP-locked SPR, the emission intensity is locked and enhanced at the frequency of SP with the emission wavelength-angle relationship still satisfying Eq. (1). Figure 2(b) and (c) present the simulated contours of absolute value of z-component magnetic field (|Hz|) for traditional SPR (electron bunch moving under uniform PEC gratings with *l* = 228 nm) and SP-locked SPR (electron bunch moving under the structure of Fig. 1 except for uniform PEC gratings with *l* = 228 nm), respectively. (In both cases, the distance between the electron bunch and the structure is 10 nm). Figure 2(b) displays that traditional SPR emits at all directions of the upper semicircle. However, Fig. 2(c) exhibits that the emission for SP-locked SPR is enhanced and concentrated around 45° with λ = 535 nm (the refractive index of dielectric layers is 2.1).

**Figure 3:**
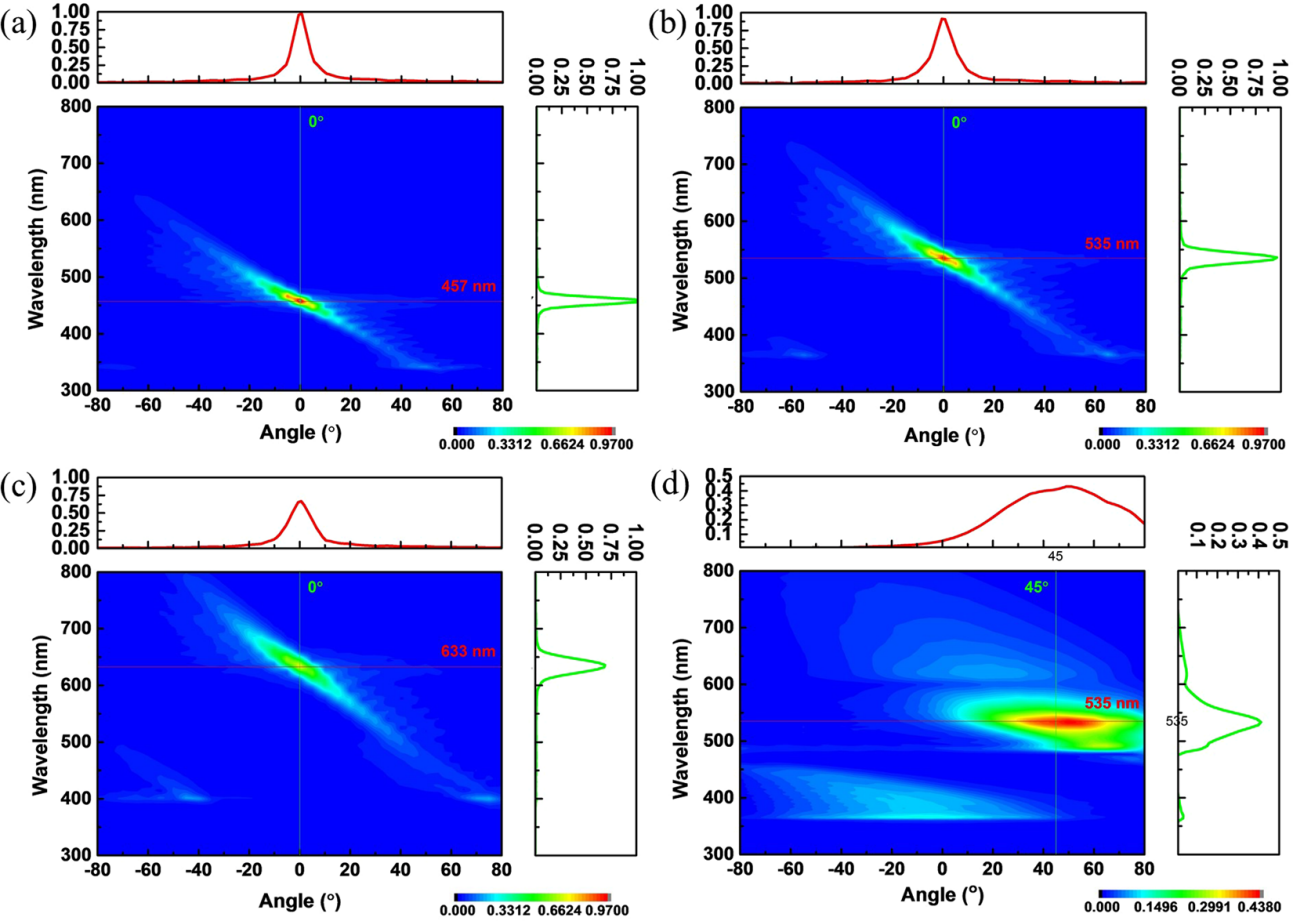
Simulated results for generation of a convergent beam with a specific wavelength. (**a**–**c**) Simulated contours of Fourier spectra of Hz fields as functions of emission wavelength and angle at the observation points for SPR emitted from the proposed structure in Fig. 1 with refractive indices of dielectric layers equal to 1.7 (λ = 457 nm), 2.1 (λ = 535 nm), and 2.6 (λ = 633 nm), respectively. Upper insets in (**a**) ((**b**), **c**(**c**)): Hz-field intensity versus emission angle at λ = 457 nm (λ = 535 nm, λ = 636 nm). Right insets in (**a**–**c**): Hz-field intensity versus emission wavelength at θ = 0°. The detailed values of *l* for chirped gratings are listed in Table 1. (**d**) The same simulated contours as in (**a**–**c**) except for uniform PEC gratings with *l* = 228 nm (i.e. Figure 2(c)). Upper and right insets in (**d**): Hz-field intensity versus emission angle at λ = 535 nm and Hz-field intensity versus emission wavelength at θ = 45°, respectively.

Next, the generation of a convergent beam with a specific emission wavelength by using the SP-locked SPR is investigated. Figure 3(a),(b) and (c) plot the simulated contours of Fourier spectra of Hz fields as functions of emission wavelength and angle at the observation points (see Fig. 1(b)) for the emission wavelength designed at 457 nm, 535 nm and 633 nm, respectively. (The geometrical parameters for the chirped gratings are given in Sec. 2). Figure 3(a),(b) and (c) show that the convergent spots are formed in the wavelength-angle plane at the designed wavelengths. And the full width at half maximum (FWHM) of emission angles (emission wavelengths) of the convergent spots for the designed wavelengths of 457 nm, 535 nm and 633 nm are 6.8° (16 nm), 8.4° (20 nm) and 9.9° (26 nm), respectively. (Since the two-dimensional simulation is performed, the real pattern in the x-z plane (*R* = 4 μm) will be a line rather than a spot). Figure 3(a–c) also display that the intensity of Hz field at the convergent spot decreases with increase of emission wavelength. These results are attributed to that for a longer wavelength, the grating period is larger and hence the grating has fewer grooves. And the larger the peak field intensity at the convergent spot is, the smaller the FWHM of angle becomes. Therefore, the FWHM of angle increases with the emission wavelength, as Fig. 3(a–c) show. Figure 3(d) presents the same contours but for the SPs-locked SPR with uniform gratings designed for λ = 535 nm (i.e. the non-convergent case, Fig. 2(c), *l* = 228 nm, emission concentrated at θ = 45°). Comparing Fig. 3(d) with Fig. 3(a–c) indicates that the Hz-field intensity at the convergent spot for the chirped gratings is about two times as large as that for the uniform gratings, which is ascribed to that the chirped gratings can emit radiation into the same place.

**Figure 4:**
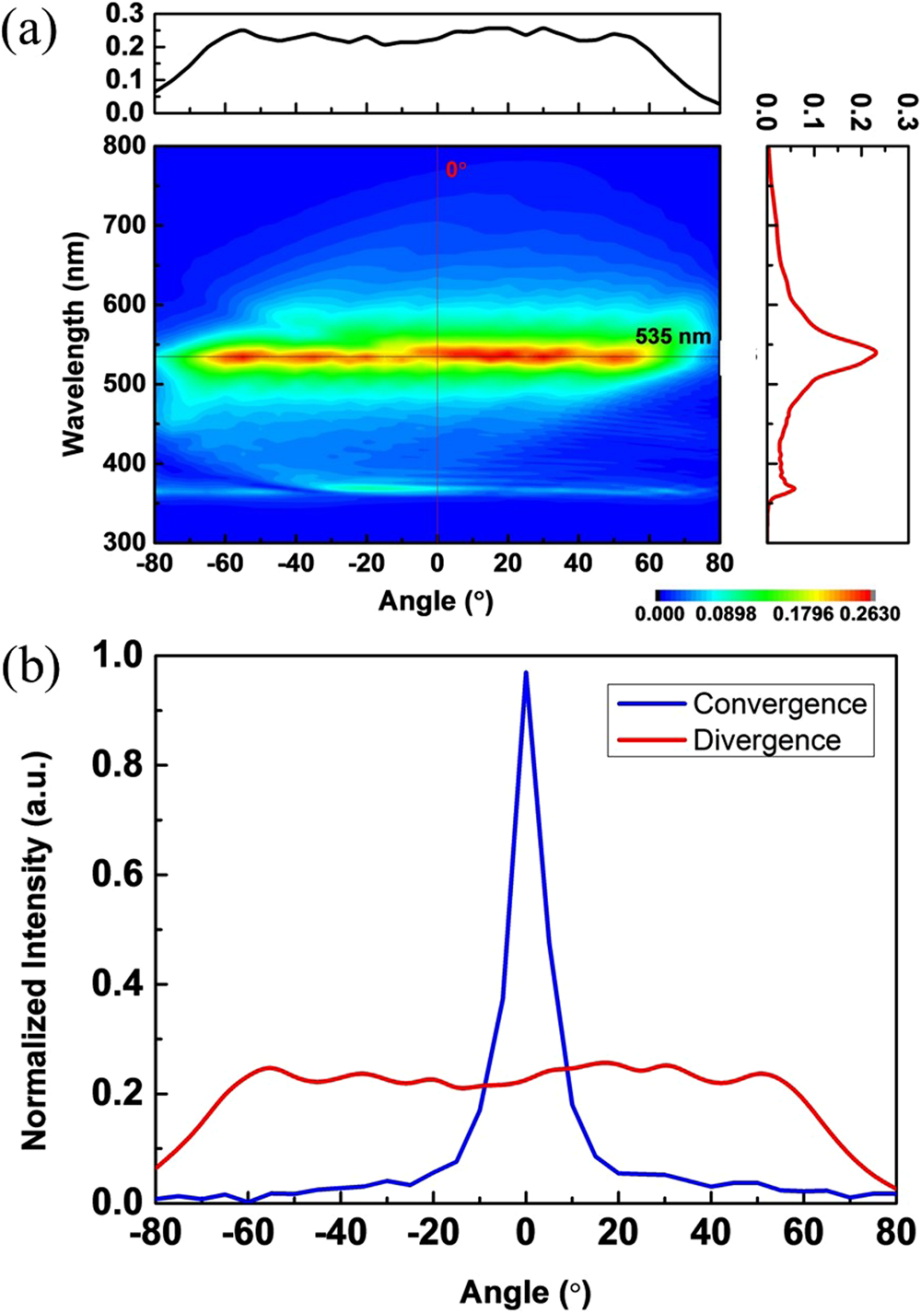
Simulated results for reversing the electron bunch moving direction. (**a**) The same simulated contours and insets as in Fig. 3(b) except for reversing the electron bunch moving direction (i.e. along the negative x-direction). (**b**) Hz-field intensities as a function of emission angle at λ = 535 nm for electron bunch moving along the positive x-direction (blue, from Fig. 3(b)) and negative x-direction (red, from Fig. 4(a)).

Subsequently, the effect of reversing the electron bunch moving direction on beam convergence is also explored. Figure 4(a) plots the simulated contours of Fourier spectra of Hz fields versus emission wavelength and angle at the observation points with the electron bunch moving along the negative x-direction. (The geometrical parameters of the device are the same as those in Fig. 3(b)). Figure 4(b) presents the field intensities of Hz fields at the observation points (see Fig. 1(b)) as a function of emission angle at λ = 535 nm for electron bunch moving along the positive x-direction (blue, a convergent SPR) and negative x-direction (red, a divergent SPR). Figure 4(a) and (b) illustrate that, when the moving direction of electron bunch is reversed, the SP-locked SPR from the proposed structure becomes divergent with the emission angle ranging from −65° to 65° for λ = 535 nm. According to Eq. (1), reversing the electron moving direction will change the sign of emission angle. As a result, the original design for converging beam becomes to diverge it. Furthermore, since the emission energy of the divergent beam is distributed over a large angular range, its peak field intensity is much smaller than that of the convergent beam in the convergent spot. Figure 4(a) and (b) imply that a light emitted from the proposed structure can be switched from a convergent beam to a divergent beam by only reversing the electron bunch moving direction.

**Figure 5:**
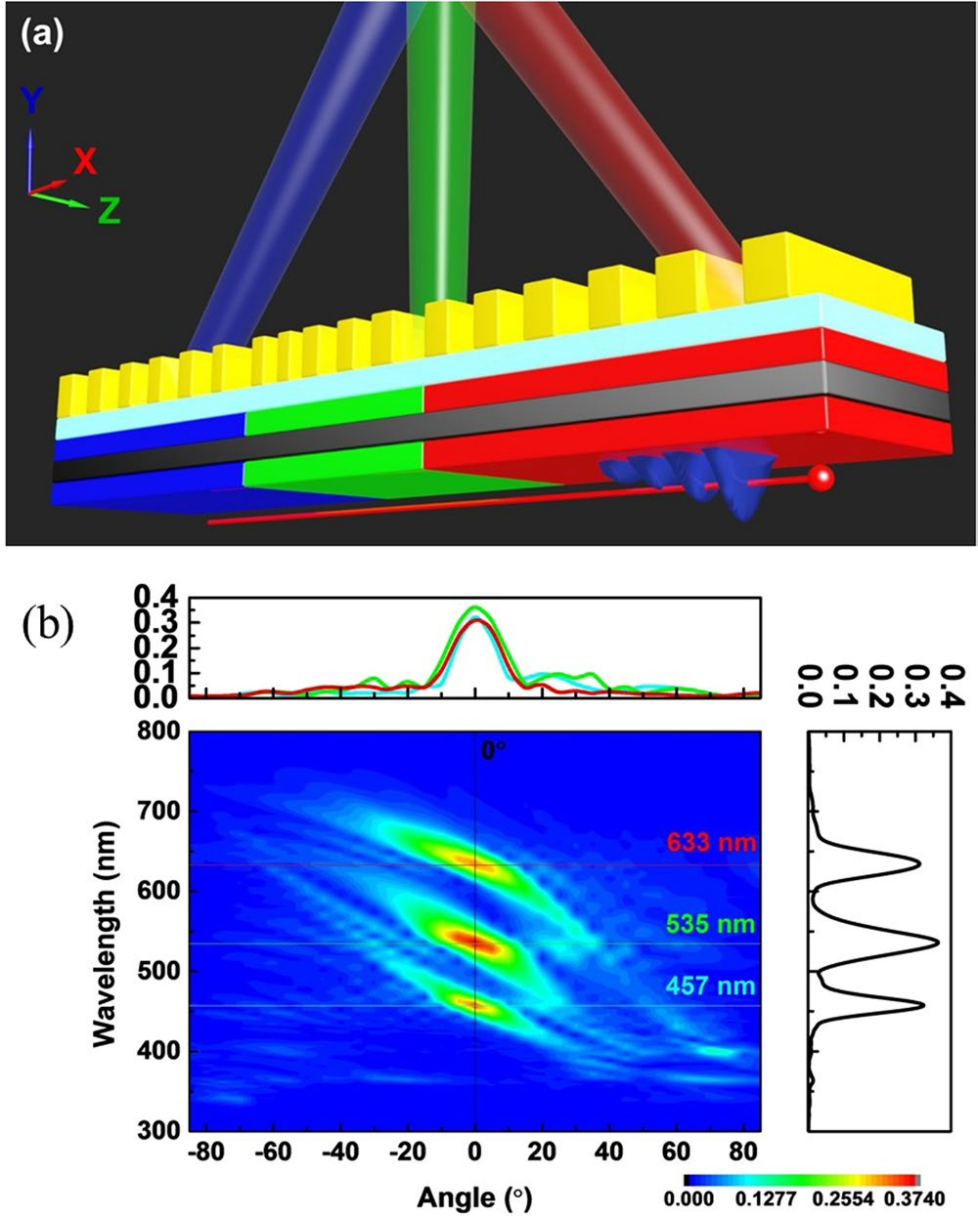
Schematic diagram and simulated results for generating a convergent beam containing multiple wavelengths. (**a**) Schematic diagram for a convergent beam involving red, green and blue lights generated by SP-locked SPR with chirped gratings on an IMI substrate. The detailed values of *l* of chirped gratings for each color are given in Table 2. (**b**) Simulated contours of Fourier spectra of Hz fields as functions of emission wavelength and angle at the observation points for SPR emitted from the structure in (**a**). Upper and right insets in (**b**): Hz-field intensity versus emission angle at λ = 457 nm (blue), 535 nm (green) and 633 nm (right) and Hz-field intensity versus emission wavelength at θ = 0° (black), respectively.

Finally, the generation of a convergent beam containing multiple wavelengths by using the SP-locked SPR is examined. Figure 5(a) plots the schematic diagram for this goal involving red, green and blue lights by using an IMI substrate with chirped gratings on it. In Fig. 5(a), the IMI substrate is divided into three sections with the refractive indices of dielectric layer, from left to right (i.e. along the x-direction), equal to 1.7 (blue light, 457 nm), 2.1 (green light, 535 nm) and 2.6 (red light, 633 nm). To form a convergent spot at θ = 0° and *R* = 6 μm (in this case, the convergent spot is designed at *R* = 6 μm), the chirped gratings need to be designed such that different lights emitted from different positions all propagate toward the convergent spot. The detailed geometrical parameters of the chirped gratings for this objective are given in Table 2 (see Method and Materials). Figure 5(b) presents the simulated contours of Fourier spectra of Hz fields as functions of emission wavelength and angle at the observation points (*R* = 6 μm). Figure 5(b) shows that the lights of 457 nm, 535 nm and 633 nm are all converged at θ = 0° with the FWHM of angles to be 13.2°, 15.7° and 16.4°, respectively (the FWHM of wavelengths for the three designed wavelength are equal to 21 nm, 34 nm and 30 nm, respectively). In our studies, the FWHM of angles of the multi-wavelength spot is larger than that of the single-wavelength spot. These results are also due to the multi-color design having fewer grooves of the chirped gratings as well as a larger value of *R* and hence smaller field intensity at the convergent spot than the single-color design. As we mentioned above, the intensity of Hz field is inversely proportional to the wavelength. Moreover, the intensity of Hz field decreases with increasing the distance between the gratings and the convergent spot. These two factors are considered in design of geometrical parameters (Table 2). Therefore, the intensities of the three wavelengths are almost the same in Fig. 5(b). However, the intensity for each wavelength component is about one third of that for the single-wavelength cases in Fig. 3. Notably, the convergent spot position (*R*) can be manipulated by changing the whole sizes of gratings and each groove period.

**Table 2:** Designed periods of chirped gratings for generation of a convergent beam involving red, green and blue lights.

Region	Designed values (*l*) of groove period (along positive x-direction) (nm)
Blue region	188, 186, 183, 181, 178, 175, 172, 169, 167, 164 (the first section, total 10 values)
Green region	185, 181, 177, 174, 171, 168 (the second section, total 6 values)
Red region	193, 190, 187, 184, 182, 180, 178, 176, 174, 173, 172 (the third section, total 11 values)

The power conversion efficiency for the proposed device is also calculated. Here we consider the proposed structure with five uniform gratings designed for the vertical emission (i.e. θ = 0°). The power conversion efficiency of SPR is defined as $\eta =\frac{{P}_{SPR}}{{P}_{0}}$, where *P*_*SPR*_ denotes the total Poynting power of SPR integrated over all the simulation time and over the whole x-space measured at 4000 nm above the electron bunch and *P*_0_ is the same Poynting power except that the structure is removed and measured at 20 nm above the electron bunch (i.e. the total available power of electron bunch in its entire path). The calculated power conversion efficiencies for blue (*l* = 150 nm), green (*l* = 176 nm) and red (*l* = 208 nm) lights are 1.34%, 1.58% and 1.15%, respectively. Moreover, the power conversion efficiency increases linearly with the number of gratings. This work offers potential applications in the fields of optical imaging, optical beam steering, holography, microdisplay, cryptography and light source.

## Discussion

In conclusion, the generation of a convergent beam via using the mechanism of SP-locked SPR is proposed and investigated by FDTD simulations. The proposed structure is composed of an IMI substrate with chirped gratings on the substrate. Based on the relationship of emission wavelength and angle of SPR, the chirped gratings are designed such that a convergent beam containing a single wavelength is formed directly above the gratings (*R* = 4 um and θ = 0°) as an electron bunch passes beneath the substrate. The wavelength of the convergent beam changes with the refractive index of dielectric layer of the IMI structure, which is determined by the frequency of SP on the IMI substrate excited by the electron bunch. The FWHM of angle for all investigated wavelengths is smaller than 10° at *R* = 4 um. Moreover, reversing the direction of electron bunch will make the emitted light to switch from a convergent beam to a divergent beam. Finally, the formation of a convergent beam containing red, green and blue lights just above the chirped gratings (i.e. at *R* = 6 um and θ = 0° with the maximum angle’s FWHM to be 16.4°) is also demonstrated. To realize it, the IMI substrate is divided into three sections and each section emits one color by adjusting the refractive index of the dielectric layers. And the chirped gratings are designed such that different lights emitted from different positions all propagate toward the convergent spot. This work offers potential applications in the fields of optical imaging, optical beam steering, holography, microdisplay, cryptography and light source.

## Methods and Materials

The FDTD program Lumerical is utilized in the simulation^22^. The two-dimensional simulation is performed in the Cartesian x–y coordinate system. The dimensions of uniform grid cells in x and y directions are both set as 2 nm. The entire region is enclosed by perfectly matched layers (PMLs). The instantaneous dielectric response, plasma frequency and collision frequency of Drude model of Ag are 5.0, 1.4433 × 10^16^ rad/s and 1.4995 × 10^14^ rad/s, respectively^23^. In this work, the electron bunch is represented by a series of dipoles in its path with phase delay that is related to the electron velocity. The movement of electron bunch is achieved by sequentially switching on and off the dipoles^24^.

**Figure 6:**
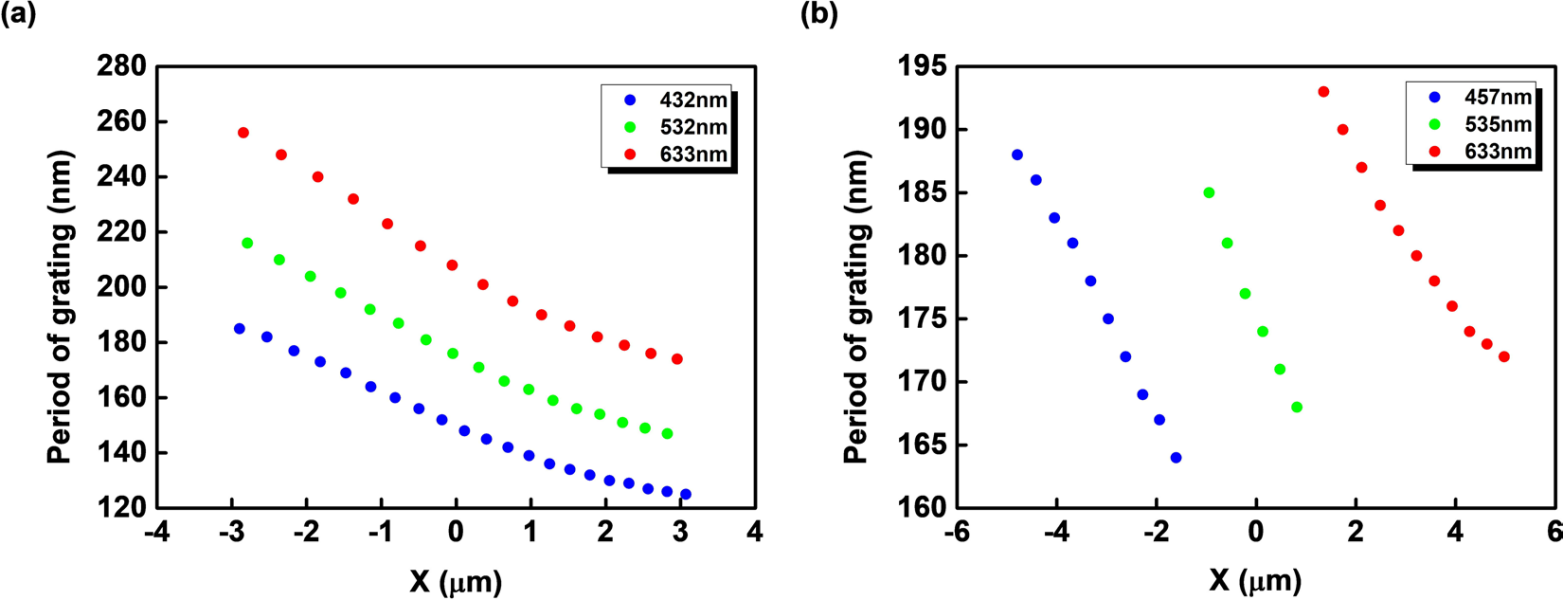
Tendencies of the grating periods. The tendencies of the grating periods (*l*) in x position for generation of a convergent beam (**a**) containing a single wavelength (Table 1) and (**b**) involving the red, green and blue lights (Table 2).

The designed periods of chirped gratings (*l*) for generation of a convergent beam containing a single wavelength and involving the red, green and blue lights are listed in Tables 1 and 2, respectively. The tendencies of the grating periods in the x position for Tables 1 and 2 are plotted in Fig. 6(a) and (b), respectively.
