# Author Correction: Generation of convergent light beams by using surface plasmon locked Smith-Purcell radiation

**DOI:** 10.1038/s41598-017-16512-0

**Published:** 2017-12-04

**Authors:** Yi-Chieh Lai, Tzu Cheng Kuang, Bo Han Cheng, Yung-Chiang Lan, Din Ping Tsai

**Affiliations:** 1 Department of Photonics and Advanced Optoelectronic Technology Center, National Cheng Kung University, Tainan, 701 Taiwan; 2 Research Center for Applied Sciences, Academia Sinica, Taipei, 115 Taiwan; 3 Department of Physics, National Taiwan University, Taipei, 10617 Taiwan

*Scientific Reports* **7**:11096; doi:10.1038/s41598-017-11622-1; Article published online 11 September 2017

The Author Contributions section in this Article is incorrect.

“Y. C. Lai, T. C. Kuang, B. H. Cheng and Y. C. Lan jointly conceived the idea. Y. C. Lai and T. C. Kuang designed and performed the calculations. Y. C. Lan and D. P. Tsai assisted in the analyzing and discussion of the results. Y. C. Lai, Y. C. Lan and D. P. Tsai prepared the manuscript. Y. C. Lan and D. P. Tsai supervised and coordinated all the work. Y. C. Lai and T. C. Kuang contributed equally to this work. All authors commented on the manuscript”.

should read:

“Y. C. Lai, T. C. Kuang, B. H. Cheng and Y. C. Lan jointly conceived the idea. Y. C. Lai and T. C. Kuang designed and performed the calculations. Y. C. Lan and D. P. Tsai assisted in the analyzing and discussion of the results. Y. C. Lai, Y. C. Lan and D. P. Tsai prepared the manuscript. Y. C. Lan and D. P. Tsai supervised and coordinated all the work. All authors commented on the manuscript”.

